# Factors associated with adherence to gluten-free diet among celiac patients in Palestine: a cross-sectional study

**DOI:** 10.1038/s41598-026-49948-4

**Published:** 2026-04-24

**Authors:** Lana Saed, Manal Badrasawi, Mira Braik, Amneh Dibas, Masa Maqboul, Qusay Abdoh, Moath Hattab

**Affiliations:** 1 Department of Medicine, Faculty of Medicine and Allied Medical Sciences, An-Najah National University, Old Campus St. 7, Nablus, Palestine; 2 Department of Nutrition and Food Technology, An-Najah National University, Nablus, Palestine; 3 Clinical Research Unit, Gastrointestinal (GI) Medical Center, Nablus, Palestine; 4 Department of Gastroenterology, Faculty of Medicine and Allied Medical Sciences, An-Najah National University, Old Campus St. 7, Nablus, Palestine

**Keywords:** Diseases, Health care, Medical research, Risk factors

## Abstract

Celiac disease (CD) is an autoimmune disease. Since the only treatment is a lifelong strict gluten-free diet (GFD), good adherence is the cornerstone. No research has examined the degree of GFD adherence among CD patients in Palestine and how it impacts the disease activity and prognosis. Therefore, this study aims to determine the level of adherence to GFD among adult patients with (CD) in Palestine, and its association with disease activity, mental health, patients’ awareness, attitude, barriers, lifestyle, and sociodemographic variables. The study involved 137 patients with celiac disease using a cross-sectional design. Participants were recruited from private and governmental clinics, as well as community-based lists and associations. Disease activity was measured using the Celiac Symptom Index (CSI), mental health was measured using the General Health Questionnaire (GHQ-12), and knowledge, awareness, attitudes, and barriers were assessed using a structured questionnaire developed by the research team. Adherence level was measured through the participants’ practices section of the questionnaire. A multivariate logistic regression was performed. The mean age of participants was 37.7 ± 13.1 years, and the majority were female (71%). Overall, 36.5% of participants demonstrated high adherence, 61.3% moderate adherence, and 2.2% low adherence. Bivariate analysis showed that symptom burden (CSI), psychosocial distress (GHQ-12), subjective knowledge, attitudes, and barriers related to meal preparation and social situations were significantly associated with adherence (*P* < 0.05). However, multivariate analysis identified a more positive attitude toward a gluten-free diet as the most consistent independent predictor of higher adherence, while the association with male sex was weaker and less stable across sensitivity analyses. A more positive attitude toward a gluten-free diet was the most consistent independent correlate of higher adherence, while the association with male sex was weaker and less stable across sensitivity analyses. These findings underscore the central role of attitudinal factors in shaping adherence and support the development of targeted, culturally sensitive interventions to optimize outcomes in patients with celiac disease.

**Supplementary Information:**

The online version contains supplementary material available at 10.1038/s41598-026-49948-4.

## Introduction

Celiac disease (CD) is an autoimmune disorder characterized by chronic inflammation of the small intestine triggered by the ingestion of gluten in genetically predisposed individuals^1^. The presence of HLA-DQ2 and/or HLA-DQ8 variants is necessary for the development of CD. These genes facilitate the presentation of gluten-derived peptides to CD4 + T cells, initiating an immune response against deaminated gluten and the enzyme transglutaminase^2^. This immune-mediated reaction leads to damage of the intestinal villi, resulting in several intestinal and extra-intestinal symptoms^1^.

Some epidemiological studies showed that the prevalence of celiac disease has been underestimated, affecting not only Europeans but also the populations of Mediterranean states. Compared to Western states (≈ 1%), many Middle Eastern prevalence estimates are similar (≈ 0.5–1.5%) and, in some studies, even higher, which may be explained by dietary behaviors such as excessive consumption of barley and wheat, as well as genetic factors^3,4^. Even though Palestine is a Mediterranean state and, in theory, might have a high incidence of CD, there is a lack of published data on CD patients. However, the number of people diagnosed with celiac disease in Palestine has risen^5^. This increase is linked by health officials to conditions of extreme stress, severe food insecurity, and a necessary dependence on inadequate, canned, and processed foods. These circumstances are recognized as environmental triggers that can activate immune system disorders in individuals with an underlying genetic predisposition to autoimmune diseases, including celiac disease^6^. This concerning trend highlights the urgent importance of investigating and understanding the specific challenges related to adherence to a gluten-free diet within the Palestinian context.

Although the term “gluten” technically refers to the storage proteins in wheat, rye, and barley, a strict lifelong gluten-free diet (GFD) excludes these grains. Pure, uncontaminated oats are tolerated by most, but not all, individuals with celiac disease, and may be included with caution^7^. A strict lifelong Gluten-Free Diet (GFD) remains the mainstay of treatment for Celiac Disease. However, adherence rates to a gluten-free diet (GFD) among adults with celiac disease remain suboptimal. Earlier studies reported a wide range of adherence, from 23% to 98%, depending on the population and assessment method^8^. More recent systematic reviews highlight that adherence is highly variable and influenced by differences in measurement tools as well as patient attitudinal and social factors^9^. This underscores the need to better understand determinants of GFD adherence to design effective interventions.

Several studies have reported many barriers to adherence to GFD, such as lack of awareness about GFD (19%), inadequate financial resources (27.2%), and non-availability of GF-food products (48.6%), lower knowledge of CD (35%); restaurant/supermarket shopping (30%); poor patient education from practitioner (17.5%); and low intention/motivation to adhere to a GFD (17.5%)^10,11^.

After a thorough review of the literature, we found no published research that examines the degree of GFD adherence among CD patients in Palestine and how it impacts the disease activity and prognosis. Furthermore, little information is known on the factors and obstacles associated with GFD adherence among CD patients in this population. This study examines GFD adherence and its determinants in the Palestinian setting. The findings may provide evidence to inform clinical practice and future educational interventions aimed at improving outcomes among adults with celiac disease.

This study aims to determine the level of adherence to a gluten-free diet among adult patients with celiac disease in Palestine and to examine its association with disease activity, mental health, patients’ awareness, attitudes, lifestyle factors, and sociodemographic characteristics. Specifically, the study seeks to assess the level of awareness about GFD among adult CD patients and identify the personal, economic, and social barriers that may hinder adherence to a GFD.

## Methods

### Study design and setting

A cross-sectional design was utilized in the present study. Data collection was carried out by administering an online questionnaire using Google Forms. Participants were recruited from private and governmental clinics, as well as from lists of beneficiaries of social assistance programs and members of relevant community associations. The process took place from August 2025 to October 2025.

### Participants

Participants were selected using convenience sampling. The study included adult patients aged ≥ 18 years from the West Bank, Palestine, with a confirmed diagnosis of celiac disease based on positive serological testing and biopsy-proven mucosal changes obtained through upper gastrointestinal endoscopy. Eligible participants were those willing to participate and able to complete the study questionnaire accurately. Exclusion criteria included patients with additional chronic diseases impacting overall health (such as inflammatory bowel disease, chronic kidney disease, or chronic liver disease), individuals recently diagnosed with cancer or receiving chemotherapy, those with diagnosed psychiatric disorders, and participants who provided incomplete responses or refused consent.

### Sample size

The sample size was calculated using the standard formula for prevalence studies: n = Z² × P (1 − P)/d², where n represents the required sample size, Z is the Z-statistic corresponding to the desired confidence level, P is the estimated prevalence of the outcome of interest, and d is the acceptable margin of error (precision). A 95% confidence level was assumed, corresponding to a Z-value of 1.96. The estimated prevalence of adherence to a gluten-free diet (GFD) among patients with celiac disease was taken as 53.2%, based on a previous study by Rajpoot et al. (2015). The margin of error was set at 10%. Based on these parameters, the calculated minimum sample size was 106 participants. To account for an anticipated non-response rate of 20%, the final required sample size was increased to 137 patients.

### Ethical consideration

The study protocol has been approved by the Internal Review Board (IRB) Committee at AN-Najah National University, which has a reference number of Agr. Vmed. Dec. 2024/34. In addition, permission and approval were obtained from the Palestinian Ministry of Health, while written consent was obtained from each participant. This study was conducted in accordance with the Declaration of Helsinki^12^.

### Data collection tool and variables

**Fig. 1:**
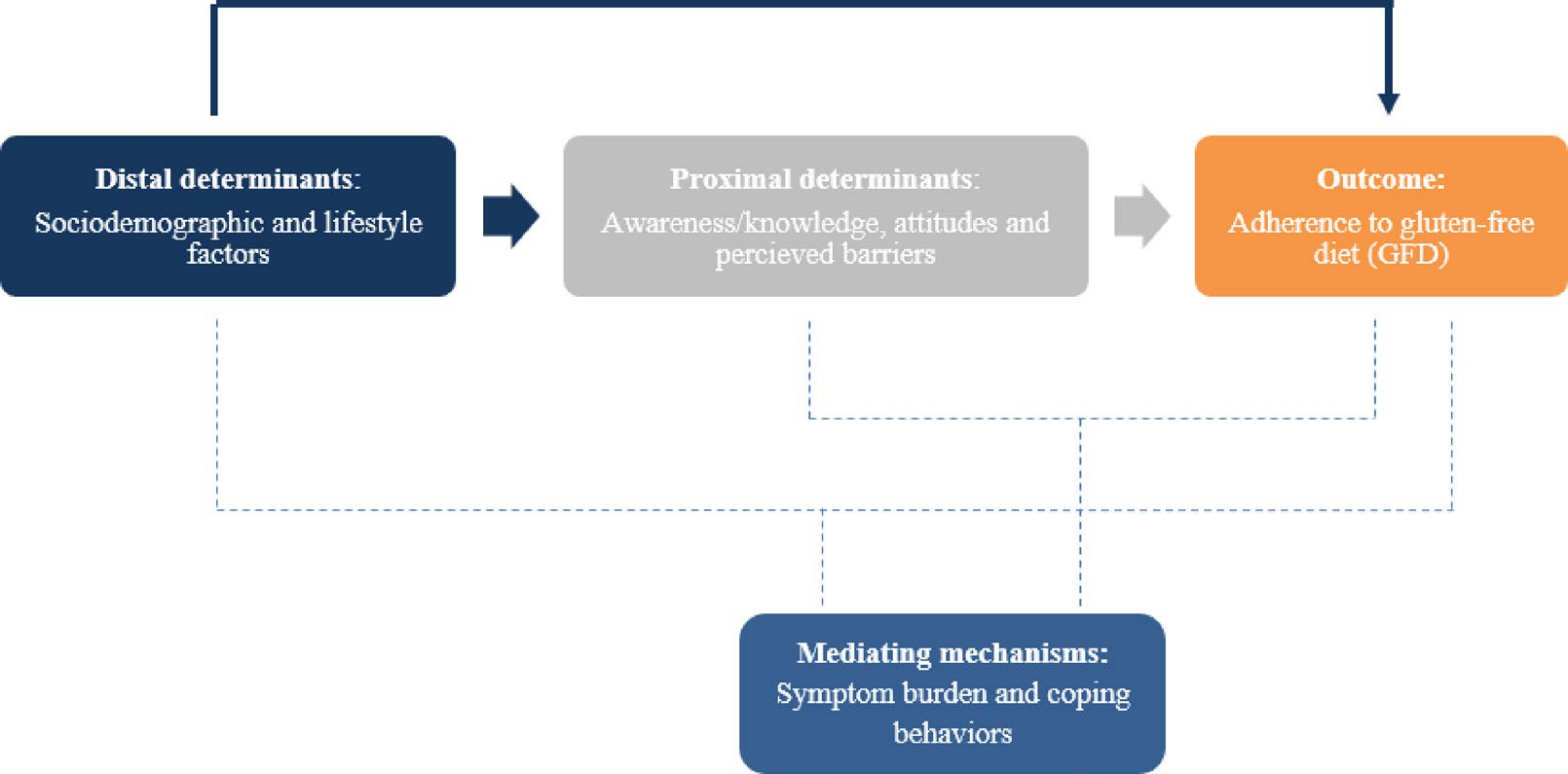
Conceptual model of determinants of adherence to a gluten-free diet.

A structured questionnaire consisting of seven sections was developed and administered using Google Forms. The sections addressed sociodemographic, disease-related information (including age at diagnosis, duration of illness, presenting symptoms before the diagnosis, possible comorbidities, dietitian and doctor follow ups, family members with a diagnosis of celiac disease, including their relation to the participant), disease activity, adherence to a gluten-free diet (GFD), barriers to adherence, lifestyle-related factors (including smoking status, sleep patterns, and physical activity), and mental health. The dependent variable in this study is adherence to a GFD, with disease activity, mental health, self-assessed knowledge, awareness, attitudes, and barriers serving as independent variables, and sociodemographic and lifestyle characteristics considered background variables. Item non-response was assessed for all questionnaire items. No missing data were observed, and therefore, complete-case analysis was performed, including all participants. (Supplementary file 1). A conceptual framework that guides the relationships among variables is presented in Fig. 1.

### Disease activity

A validated self-administered questionnaire, the Celiac Symptom Index (CSI), was used to assess symptom intensity in patients with celiac disease. It consists of 16 items assessing domains related to CD-specific symptoms (11-items) and general health (5-items) rated via a 5-point Likert scale, giving a total score ranging from 6 to 80. A total score of < 30 was classified as low symptom burden (suggestive of minimal symptoms or clinical remission), scores between 31 and 44 reflected moderate symptom burden, whereas scores ≥ 45 indicated a high symptom burden consistent with active or poorly controlled disease. Cronbach’s alpha (α) value = 0.875^13^.

### Adherence to GFD assessment

Adherence to a gluten-free diet (GFD) was assessed using the practices domain of a research team–developed questionnaire. The overall questionnaire included four domains: self-assessed knowledge (4 items; total score 4–20), awareness (6 items; maximum score 21), attitudes toward the GFD (6 items; total score 6–30), and practices. For the assessment of dietary adherence, only the practices domain was used, as practice-based measures more directly reflect real dietary behavior than knowledge or attitude alone. The full questionnaire and scoring details are provided in Supplementary File 1.

The practices domain included items addressing routine gluten-free dietary behaviors and potential sources of intentional or unintentional gluten exposure. Item scores were summed to generate a total practice score, which was converted to a percentage of the maximum possible score. The maximum possible practices score was 21 points. Accordingly, adherence categories corresponded to the following raw score ranges: high adherence = 19–21/21 (90.5%–100%), moderate adherence = 16–18/21 (76.2%–85.7%), and low adherence = 0–15/21 (0%–71.4%). For inferential analyses, moderate and low adherence were combined and compared with high adherence.

The questionnaire was developed with reference to existing literature and adherence assessment approaches in celiac disease, then refined through expert review to improve clarity and contextual relevance. However, it was not formally validated against external reference standards such as dietitian-led assessment, serologic markers, urinary gluten immunogenic peptides, or previously validated adherence tools. Therefore, the practices-based score was used as a pragmatic proxy for GFD adherence, and the findings were interpreted with appropriate caution.

### Barriers

Barriers to adherence were assessed in two domains: access/availability barriers (9 items) and social barriers (7 items), each rated on a 3-point Likert scale. For each participant, a mean item score was calculated for each domain, yielding a score from 1 to 3, with higher values indicating greater perceived barriers. In the primary inferential analyses, these barrier scores were analyzed as continuous variables. For descriptive presentation only, the mean scores were additionally grouped into low (< 1.66), moderate (1.66–2.33), and high (> 2.33) categories. These descriptive categories were not intended to represent clinically validated thresholds.

### Mental health

The general health questionnaire is a 12-item validated and reliable tool for general screening to measure minor psychological distress. Likert scoring was used for the rating scale in this study, which ranges from 0 to 3, where zero represents the healthiest and 3 represents poor health/illness, and the total score can range from 0 to 36. (Cronbach’s alpha = 0.87)^14–18^.

The first draft of the questionnaire was revised, modified, and edited by the research supervisors before sending it to six experts in the subject to ensure content validity. All expert comments were considered. A pilot study was conducted on 20 patients with celiac disease to assess the internal consistency of the questionnaire domains. Cronbach’s alpha (α) was calculated separately for each subscale. Reliability was interpreted as follows: <0.60 poor, 0.60–0.69 questionable, 0.70–0.79 acceptable, 0.80–0.89 good, and ≥ 0.90 excellent. The internal consistency coefficients were as follows: self-assessed knowledge (α = 0.81), awareness (α = 0.84), attitudes (α = 0.86), and barriers (α = 0.80). The overall scale demonstrated good internal consistency (α = 0.82).

### Statistical analysis

Data were summarized as mean ± standard deviation for continuous variables and frequency (percentage) for categorical variables. Bivariate associations with adherence group were examined using appropriate statistical tests according to variable type. Multivariable logistic regression was performed to identify independent predictors of high adherence versus moderate/low adherence. Because numerous bivariate comparisons were performed, these findings were interpreted cautiously as exploratory because of the risk of type I error. To evaluate the logistic regression model, multicollinearity was assessed using variance inflation factors (VIFs), calibration using the Hosmer–Lemeshow goodness-of-fit test, and discrimination using the area under the receiver operating characteristic curve (AUC). Sensitivity analyses included alternative adherence cut-points, a reduced multivariable model, and re-analysis of ordinal variables using trend-type approaches where appropriate. A two-sided p-value < 0.05 was considered statistically significant. Analyses were conducted using R version 4.5.2.

## Results

**Fig. 2:**
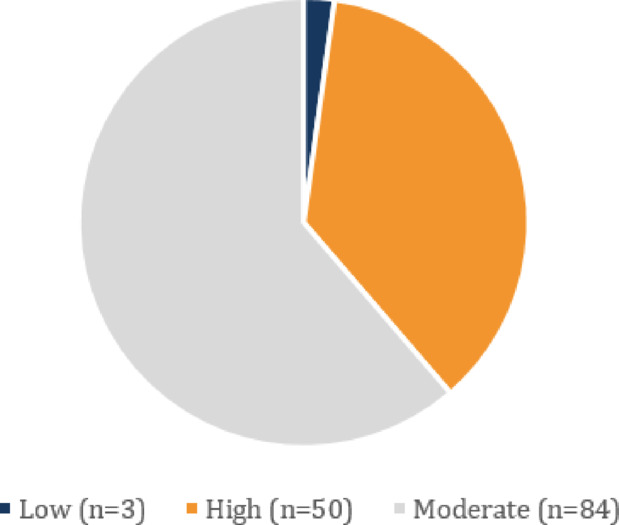
Adherence rates by category.

A total of 137 adults with biopsy/serology-confirmed celiac disease were included. Adherence was categorized according to the practices score as high (19–21/21; 90.5%–100%), moderate (16–18/21; 76.2%–85.7%), and low (0–15/21; 0%–71.4%). Based on this definition, 50 (36.5%) participants were classified as High adherence, 84 (61.3%) as Moderate, and 3 (2.2%) as Low. For inferential analyses, Moderate and Low were combined (Moderate/Low, *n* = 87) and compared against High (*n* = 50) (Fig. 2).

**Fig. 3:**
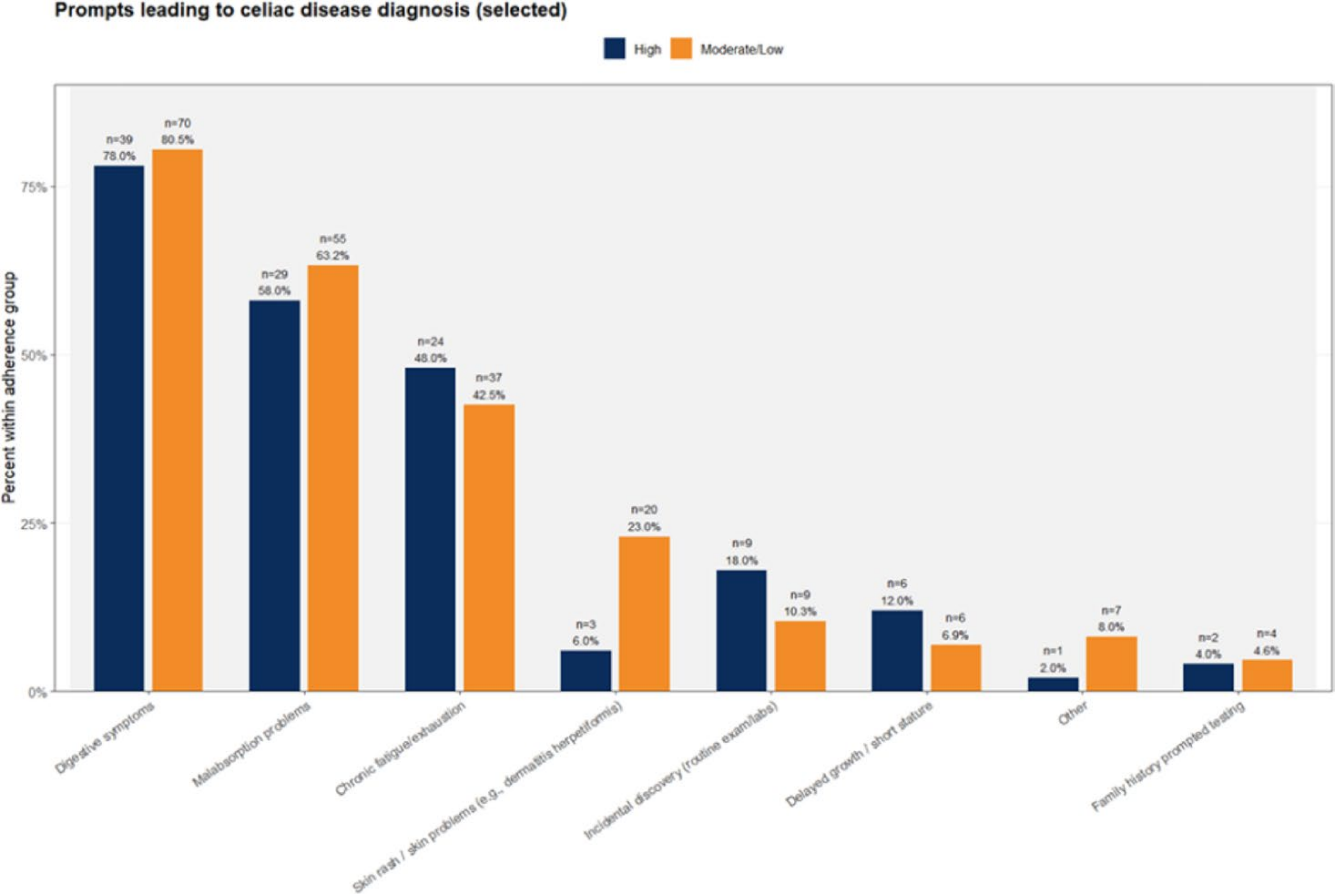
Prompts leading to celiac disease diagnosis (selected).

The mean age was 37.7 ± 13.1 years, and most participants were females (71%). The average years since diagnosis was 10.8 ± 9.33. Of the patients, 15 (11%) were newly diagnosed (with a duration less than two years), 37 (27%) had an established/intermediate duration (with a duration of 2–5 years), and 85 (62%) had a long-term diagnosis (with a duration of more than five years). Presenting symptoms that warranted the diagnosis were distributed as digestive symptoms (79.56%), malabsorption problems (61.3%), chronic fatigue/exhausted (44.5%), skin rash/skin problem (e.g. dermatitis herpetiformis), incidental discovery through routine labs (13.1%), delayed growth/short stature (8.76%), family history of celiac disease (4.4%), the remaining percentage (5.84%) reported to have other reasons for the diagnosis such as: through screening tests for autoimmune diseases in diabetes mellitus patients, complaining of symptoms related to anemia, recurrent oral ulcers and shortness of breath (Fig. 3). 39 (28%) were compliant with regular yearly doctor follow-up. However, only 15 (11%) of the patients followed up regularly with nutritionist visits.

There were no statistically significant differences between adherence groups in sex (*P* = 0.458), age (*P* = 0.747), place of residence (*P* = 0.601), marital status (*P* = 0.198), employment status (*P* = 0.101), educational level (*P* = 0.922), or income (*P* = 0.118) (Table 1). Neither was there any correlation with smoking status (*P* = 0.619), age at diagnosis (*P* = 0.417), years since diagnosis (*P* = 0.656), or years since diagnosis categories (*P* = 0.931). Regular doctor or nutritionist follow-ups were also not significant (*P* = 0.782) (*P* = 0.560), respectively. Sleep duration distributions were comparable between groups (*P* = 0.243) Table 1.

In contrast, several symptoms and psychosocial measures differed by adherence status. Participants with Moderate/Low adherence had higher symptom burden as reflected by higher CSI total scores (45.08 ± 10.15 vs. 41.10 ± 12.55; *P* = 0.045) Table 2. Psychological distress (GHQ-12) was also higher in the Moderate/Low group (27.09 ± 5.35 vs. 24.88 ± 3.96; *P* = 0.009). Self-assessed knowledge scores were modestly higher among participants with High adherence (range 4–20): 17.08 ± 2.72 vs. 16.09 ± 2.71; *P* = 0.043; percentage: 85.40 ± 13.58 vs. 80.46 ± 13.55; *P* = 0.043), However, as for the total awareness score, which reflected results of the standardized GFD knowledge quiz, the participants exhibited similar scores with a mean of (16.60 ± 2.12 vs. 15.87 ± 2.73; *P* = 0.149), resulting in a non-significant relationship between patients’ objective knowledge and adherence Table 3.

**Fig. 4:**
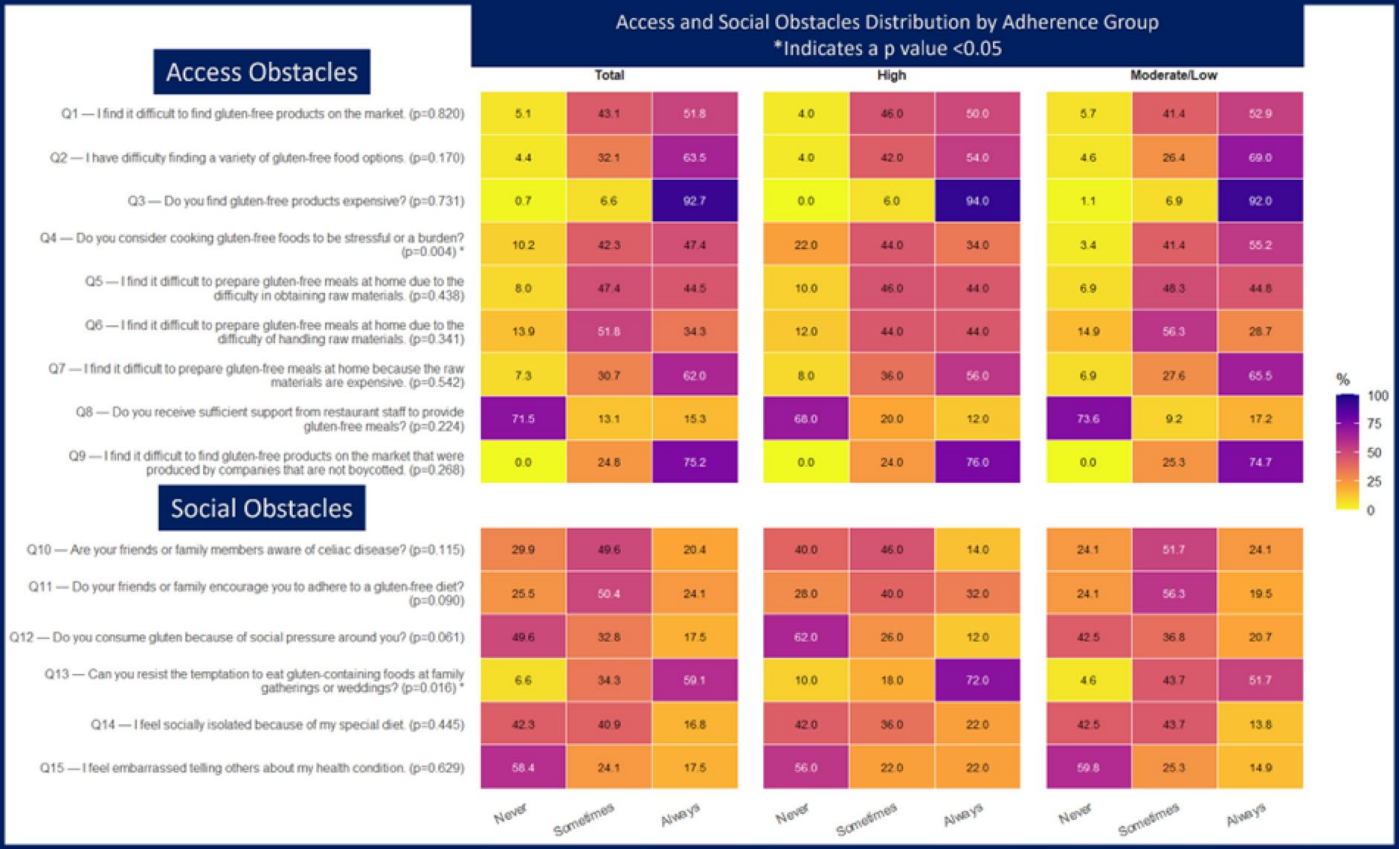
Access and social obstacles distribution by adherence group.

**Fig. 5:**
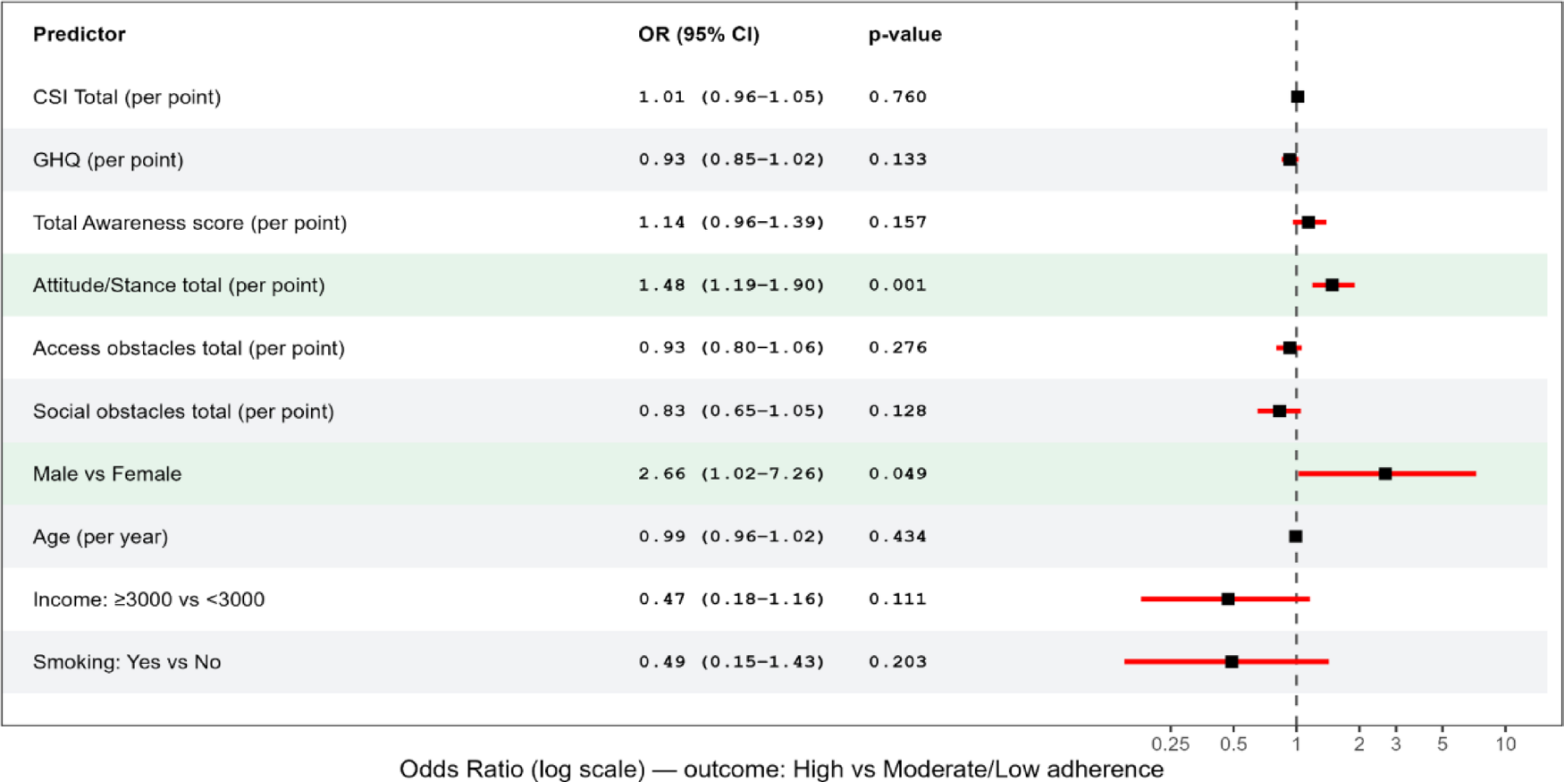
Multivariable logistic regression model (high adherence vs. moderate/low).

Attitude/stance distributions differed between groups (*P* = 0.048), with a larger proportion of the High-adherence group achieving the maximum score of 30 (54% vs. 32%) Table 3. The attitude most strongly associated with adherence was the desire to avoid health complications from gluten consumption, which showed the largest mean difference between adherence groups and a highly significant association (*P* < 0.001). Followed by the urge to maintain long-term health (*P* < 0.001), symptom relief (*P* = 0.002), and general belief in the importance of the diet (*P* = 0.002), while social support and perceived symptom control were not significantly associated with adherence (Figs. 4,  5).

When barriers were examined in this sample, 92 (67%) participants reported facing high access-barrier scores. An additional 42 (31%) reported moderate access-barrier scores, while only 3 participants experienced low access-barrier scores (2.2%) Table 3 Comparison of *mental health*,* self*-assessed knowledge, *a*wareness, *a*ttitudes *and b*arriers with adherence group*s*. While overall access and social barrier scores were not different between groups, specific psychological and social challenges emerged as significant. Notably, higher adherence was significantly associated with not finding cooking to be a stressful task (*P* = 0.004). In contrast, a lower adherence rate was associated with lower resistance to eating gluten-containing foods at a family gathering or wedding (*P* = 0.016) (Table 4).

Because numerous bivariate comparisons were performed, the risk of type I error was considered. After accounting for multiple comparisons across the reported bivariate tests, the total attitude/stance score remained the most robust association, whereas other nominally significant findings were interpreted as exploratory.

**Table 1:** Sociodemographic distribution by adherence groups.

Variable	Overall (*N* = 137)	High (*N* = 50)	Moderate/Low (*N* = 87)	*p*-value
**Sex**				
**Female**	97 (71%)	33 (66%)	64 (74%)	0.458
**Male**	40 (29%)	17 (34%)	23 (26%)	
**Age (years)**	37.66 ± 13.12	37.22 ± 13.52	37.92 ± 12.95	0.747
**Place of residence**				
**Camp**	9 (6.6%)	2 (4.0%)	7 (8.0%)	0.601
**City**	59 (43%)	21 (42%)	38 (44%)	
**Village**	69 (50%)	27 (54%)	42 (48%)	
**Value of family income**				
**Less than 1500**	43 (31%)	19 (38%)	24 (28%)	0.118
**From 1500 to 3000**	52 (38%)	16 (32%)	36 (41%)	
**From 3000 to 5000**	25 (18%)	12 (24%)	13 (15%)	
**More than 5000**	17 (12%)	3 (6.0%)	14 (16%)	
**Marital status**				
**Married**	91 (66%)	29 (58%)	62 (71%)	0.198
**Single**	37 (27%)	18 (36%)	19 (22%)	
**Other**	9 (6.6%)	3 (6.0%)	6 (6.9%)	
**Employment status**				
**Worker/employee**	43 (31%)	17 (34%)	26 (30%)	0.101
**Unemployed**	75 (55%)	28 (56%)	47 (54%)	
**Part-time work**	15 (11%)	2 (4.0%)	13 (15%)	
**Retired**	4 (2.9%)	3 (6.0%)	1 (1.1%)	
**Educational level**				
**Primary**	8 (5.8%)	3 (6.0%)	5 (5.7%)	0.922
**Secondary**	38 (28%)	14 (28%)	24 (28%)	
**University/Diploma**	83 (61%)	31 (62%)	52 (60%)	
**Postgraduate studies**	8 (5.8%)	2 (4.0%)	6 (6.9%)	

**Table 2:** Comparison of disease characteristics and lifestyle factors with adherence groups.

Variable	Overall (*N* = 137)	High (*N* = 50)	Moderate/Low (*N* = 87)	*p*-value*
**Disease-related information**				
**Disease duration**				
**Age at diagnosis (years)**	26.64 ± 12.41	25.16 ± 11.77	27.49 ± 12.75	0.417
**Years since diagnosis**	10.80 ± 9.33	11.28 ± 9.59	10.52 ± 9.22	0.656
**Years since diagnosis group**				
**< 2 years**	15 (11%)	5 (10%)	10 (11%)	0.931
**2–5 years**	37 (27%)	13 (26%)	24 (28%)	
**> 5 years**	85 (62%)	32 (64%)	53 (61%)	
Regular doctor follow-up (Yes vs. No)**				
**Yes**	39 (28%)	16 (32%)	23 (26%)	0.782
**No**	98 (72%)	34 (68%)	64 (74%)	
**Regular nutritionist follow-up (Yes)**	15 (11%)	7 (14%)	8 (9.2%)	0.560
**Smoking status**				
**Yes**	(%20.4) 28	8 (16.0%)	20 (23.0%)	0.619
**No**	104 (75.9%)	40 (80.0%)	64 (73.6%)	
**Previous smoker**	5 (3.6%)	2 (4.0%)	3 (3.4%)	
**Family history of celiac disease**				0.666
**Yes**	47 (34.3%)	16 (32.0%)	31 (35.6%)	
**No**	90 (65.7%)	34 (68.0%)	56 (64.4%)	
**Sleep hours/night**				
**2**	1 (0.7%)	1 (2.0%)	0 (0%)	0.243
**4**	6 (4.4%)	2 (4.0%)	4 (4.6%)	
**5**	21 (15%)	5 (10%)	16 (18%)	
**6**	40 (29%)	11 (22%)	29 (33%)	
**7**	39 (28%)	18 (36%)	21 (24%)	
**8**	20 (15%)	7 (14%)	13 (15%)	
**9**	6 (4.4%)	4 (8.0%)	2 (2.3%)	
**10**	3 (2.2%)	2 (4.0%)	1 (1.1%)	
**12**	1 (0.7%)	0 (0%)	1 (1.1%)	
**Symptom burden (Celiac Symptom Index)**				
**Group 1 (≤ 30)**	13 (9.5%)	8 (16%)	5 (5.7%)	0.134
**Group 2 (31–44)**	65 (47%)	23 (46%)	42 (48%)	
**Group 3 (≥ 45)**	59 (43%)	19 (38%)	40 (46%)	
**CSI total**	43.63 ± 11.20	41.10 ± 12.55	45.08 ± 10.15	0.045

**Table 3:** Comparison of mental health, self-assessed knowledge, awareness, attitudes and barriers with adherence groups.

Variable	Overall (*N* = 137)	High (*N* = 50)	Moderate/Low (*N* = 87)	*p*-value
**Mental Health (General Health Questionnaire-12)**				
**Negative (0–15)**	3 (2.2%)	1 (2.0%)	2 (2.3%)	> 0.999
**Positive (> 15)**	134 (98%)	49 (98%)	85 (98%)	
**GHQ-12 total**	26.28 ± 4.99	24.88 ± 3.96	27.09 ± 5.35	0.009
**Self-assessed knowledge group**				
**Low**	7 (5.1%)	3 (6.0%)	4 (4.6%)	0.019
**Moderate**	42 (30.7%)	8 (16.0%)	34 (39.1%)	
**High**	88 (64.2%)	39 (78.0%)	49 (56.3%)	
**Self-assessed knowledge (4–20)**	16.45 ± 2.74	17.08 ± 2.72	16.09 ± 2.71	0.043
**Awareness**				
**Awareness total (max 21)**	16.14 ± 2.54	16.60 ± 2.12	15.87 ± 2.73	0.149
**Attitude/Stance total (max 30)**				
**22**	1 (0.7%)	0 (0%)	1 (1.1%)	0.048
**23**	5 (3.6%)	0 (0%)	5 (5.7%)	
**24**	13 (9.5%)	1 (2.0%)	12 (14%)	
**25**	8 (5.8%)	1 (2.0%)	7 (8.0%)	
**26**	10 (7.3%)	3 (6.0%)	7 (8.0%)	
**27**	16 (12%)	5 (10%)	11 (13%)	
**28**	11 (8.0%)	4 (8.0%)	7 (8.0%)	
**29**	18 (13%)	9 (18%)	9 (10%)	
**30**	55 (40%)	27 (54%)	28 (32%)	
**Attitude/Stance total**	27.89 ± 2.32	28.90 ± 1.56	27.31 ± 2.49	< 0.001
**Practices**				
**Practices total (max 21)**	17.21 ± 2.56	19.66 ± 0.72	15.80 ± 2.13	< 0.001
**Barriers**				
**Access obstacles**				
**Low barriers**	3 (2.2%)	1 (2.0%)	2 (2.3%)	0.368
**Moderate barriers**	42 (31%)	19 (38%)	23 (26%)	
**High barriers**	92 (67%)	30 (60%)	62 (71%)	
**Access obstacles mean**	2.42 ± 0.34	2.38 ± 0.36	2.45 ± 0.32	0.384
**Social obstacles**				
**Low barriers**	12 (8.8%)	3 (6.0%)	9 (10%)	0.567
**Moderate barriers**	103 (75%)	40 (80%)	63 (72%)	
**High barriers**	22 (16%)	7 (14%)	15 (17%)	
**Social obstacles mean**	1.95 ± 0.31	1.93 ± 0.30	1.96 ± 0.31	0.454

**Table 4:** Categorical distribution of attitudes by adherence group.

Item	High (*n* = 50) Mean ± SD	Moderate/Low (*n* = 87) Mean ± SD	*p*-value
Q1: Do you believe that adhering to a gluten-free diet is important for your health?	4.96 ± 0.20	4.75 ± 0.46	0.002
Q2: The relief from symptoms motivates me to adhere to a gluten-free diet.	4.88 ± 0.33	4.57 ± 0.68	0.002
Q3: “Maintaining my long-term health encourages me to stick to a gluten-free diet.”	4.90 ± 0.30	4.55 ± 0.64	< 0.001
Q4: I want to avoid the health complications resulting from consuming gluten, so I adhere to the diet.	4.84 ± 0.37	4.45 ± 0.71	< 0.001
Q5: Social motivation and family support help me stick to a gluten-free diet.	4.52 ± 0.68	4.29 ± 0.87	0.144
Q6: A gluten-free diet actually helps in controlling symptoms.	4.80 ± 0.40	4.66 ± 0.48	0.074

A multivariable logistic regression model was fitted to identify independent predictors of high adherence (vs. moderate/low). Covariates included CSI total score, GHQ total score, total awareness score, attitude/stance total, access obstacles total, social obstacles total, sex, age, monthly family income (≥ 3000 vs. < 3000), and smoking status.

In the adjusted model, a higher attitude/stance total score was independently associated with higher odds of high adherence (aOR 1.48 per 1-point increase; 95% CI 1.19–1.90; *P* = 0.001). Male sex was also associated with higher odds of high adherence compared with female sex (aOR 2.66; 95% CI 1.02–7.26; *P* = 0.049).

No other covariates demonstrated statistically significant associations with High adherence. CSI total score was not associated with adherence (aOR 1.01 per point; 95% CI 0.96–1.05; *P* = 0.760). GHQ showed a non-significant inverse trend (aOR 0.93 per point; 95% CI 0.85–1.02; *P* = 0.133). Total Awareness score was not statistically significant (aOR 1.14 per point; 95% CI 0.96–1.39; *P* = 0.157). Access obstacles (aOR 0.93 per point; 95% CI 0.80–1.06; *P* = 0.276) and social obstacles (aOR 0.83 per point; 95% CI 0.65–1.05; *P* = 0.128) were also not significant. Age was not associated with adherence (aOR 0.99 per year; 95% CI 0.96–1.02; *P* = 0.434). Participants with income ≥ 3000 did not have significantly different odds of High adherence compared with those with income < 3000 (aOR 0.47; 95% CI 0.18–1.16; *P* = 0.111). Smoking status was not associated with High adherence (aOR 0.49; 95% CI 0.15–1.43; *P* = 0.203)^5^.

Given the relatively high number of covariates in relation to the number of high-adherence events, additional model diagnostics and sensitivity analyses were performed. Multicollinearity was low, with variance inflation factor values ranging from 1.07 to 1.48, indicating no important collinearity among predictors. Model calibration was acceptable according to the Hosmer–Lemeshow goodness-of-fit test (*p* = 0.625), and discrimination was acceptable (area under the receiver operating characteristic curve = 0.756).

Sensitivity analyses using alternative adherence cut-points showed that the association between attitude/stance total score and high adherence remained consistent. When high adherence was defined as ≥ 18/21, attitude/stance remained significant (OR 1.51, *p* < 0.001), and male sex remained associated with higher adherence (OR 2.86, *p* = 0.038). Under the original threshold of ≥ 19/21, attitude/stance remained significant (OR 1.48, *p* = 0.001), and male sex was borderline significant (OR 2.66, *p* = 0.049). When a stricter threshold of ≥ 20/21 was used, attitude/stance remained significant (OR 1.66, *p* = 0.004), whereas male sex was no longer associated with adherence (OR 0.70, *p* = 0.576). These findings suggest that the attitude/stance association was robust, whereas the sex association was less stable across alternative definitions.

In a reduced-model sensitivity analysis, attitude/stance total remained independently associated with high adherence (OR 1.45, *p* < 0.001), whereas male sex was no longer statistically significant (OR 2.01, *p* = 0.130).

Re-analysis of ordinal variables using trend-type approaches did not materially change the results. Neither income (OR 0.80 per category increase, *p* = 0.222) nor sleep duration (OR 1.18 per hour increase, *p* = 0.190) showed a significant linear trend with adherence.

## Discussion

This study provided important insights into factors affecting adherence to a gluten-free diet (GFD) among adults diagnosed with celiac disease through biopsy and serology in Palestine. The findings indicated that optimal adherence to a strict GFD remains challenging for many Palestinian patients. This pattern is generally consistent with reports from other regional and low- to middle-income settings, where social, economic, and healthcare-related barriers frequently interfere with long-term dietary compliance^19^.

Our descriptive findings revealed several critical and context-specific challenges to gluten-free diet adherence among Palestinian adults with celiac disease. Only 28% of participants reported seeing a doctor for follow-up at least once per year, and a mere 11% reported seeing a nutritionist, highlighting a substantial gap in multidisciplinary care and access to professional dietary guidance. These low rates of clinical follow-up likely contribute to ongoing difficulties in maintaining adherence and underscore the need for targeted health policy interventions to improve routine care. Symptom burden was also high, with 43% of participants scoring in the active disease range (CSI ≥ 45), indicating substantial unmet clinical needs and potential under-management of the condition. Psychological distress was extremely prevalent, with 98% of participants affected, likely reflecting the acute crisis context during data collection rather than stable population characteristics. While this limits generalizability to more stable periods, it also provides a unique insight into the lived experience of celiac patients navigating dietary management under extreme conditions. In addition, participants reported unique sociopolitical barriers, including instances of boycotting that limited access to gluten-free foods or restricted participation in social and commercial settings. Such context-specific obstacles reflect structural and environmental factors that are not captured in traditional adherence assessments but play an important role in shaping dietary behaviors.

In the primary adjusted model, male sex and more positive attitudes toward the gluten-free diet were associated with higher adherence. However, across sensitivity analyses, the association with attitude remained robust, whereas the association with male sex was less stable. While bivariate analyses revealed additional associations, including higher symptom burden, greater psychological distress, and higher self-assessed knowledge, these did not remain significant after adjustment, suggesting that their apparent effects may be mediated or confounded by other factors. Accordingly, our primary interpretation focuses on the multivariable findings, with bivariate associations serving as contextual observations that may help explain potential pathways influencing adherence. Because numerous bivariate comparisons were performed, these findings should be interpreted cautiously. In this context, the total attitude/stance score appeared to be the most robust bivariate correlate of adherence, whereas other nominally significant associations may be less stable.

A significant correlation between a positive attitude towards the GFD and high adherence was also observed in our sample (*P* < 0.001). Our findings also highlighted that motivation rooted in preserving long-term health seems to be more impactful than motivation driven solely by short-term symptom relief, especially the desire to prevent long-term complications from gluten exposure (*P* < 0.001). These patients have probably acknowledged that CD is not a short-term illness that will get better over time, and that ongoing treatment is necessary because of its long-lasting nature and to obtain better long-term health free of disease-related complications.

Male sex was associated with higher odds of high adherence in the primary adjusted model; however, this association was borderline and was not stable across sensitivity analyses using alternative adherence thresholds and a reduced model. Therefore, this finding should be interpreted cautiously. Prior literature on sex differences in gluten-free diet adherence is mixed, and the mechanisms underlying any sex-based differences remain uncertain. Potential explanations may include differences in perceived stress, household roles, financial autonomy, or food preparation responsibilities, but these hypotheses could not be tested in the present study^20–23^.

Severe psychosocial factors established a significant relationship with adherence in bivariate analysis (*P* = 0.009). These findings were consistent with previous literature, in which Anxiety and depression were found to be associated with lower adherence to a gluten-free diet (anxiety OR = 0.7, *P* < 0.001; depression OR = 0.5, *P* < 0.001)^24^. This may be attributed to the way depression and anxiety affect the cognitive and behavioral capacities required for dietary self-management, including motivation, self-control, and consistency with dietary instructions, making it difficult to plan meals or manage social situations involving food due to the emotional burden. Although this association was no longer apparent in the multivariate analyses after adjusting for other factors.

When comparing patients with higher symptom burden (CSI total score) to others with lower burden, moderate/low adherence was associated with higher symptom burden in bivariate analysis (*P* = 0.045). This relationship has also been reported in other studies, indicating that experiencing fewer gastrointestinal symptoms was associated with better adherence to a GFD, where adequate adherers reported significantly fewer symptoms than inadequate adherers (*P* = 0.001)^25^. However, in the adjusted model, this association became non-significant, suggesting that symptom burden alone was not an independent predictor of adherence. This suggests that gluten ingestion can affect patients differently because symptom severity depends on the level of gluten exposure and is highly individual between patients. For instance, those who experience fewer symptoms with gluten ingestion may not necessarily observe direct benefits from the GFD and thus have poorer adherence as they perceive gluten ingestion to be less harmful^26^. In summary, the relationship between symptom burden and adherence may be bidirectional and complicated to interpret. Patients with celiac disease may become trapped in a vicious cycle. When they don’t experience immediate or significant improvement from adhering to the diet, their motivation to follow the diet decreases. Consequently, adherence worsens, and the disease may further deteriorate.

Self-assessed knowledge scores were modestly higher among participants with high adherence (*P* = 0.046). This relationship reflects more than simple information retention and is best interpreted through a social-cognitive lens, specifically Bandura’s theory of self-efficacy. Self-efficacy is a principal connection between knowledge and action, since the belief that one can do a behavior usually occurs before one actually attempts the behavior^27^. Indicating that patients who rate their knowledge higher are likely expressing greater confidence in their capability to execute the complex behaviors required for strict adherence, such as distinguishing food labels, managing cross-contamination, and navigating social dining. While self-confidence in GFD knowledge was significantly associated with adherence, objective knowledge scores were not. This suggests that successful dietary management relies on a patient’s perceived capability (self-efficacy) in addition to factual knowledge.

When assessing barriers facing participants in this sample, results reflected an overall state of shortage in the accessibility of both gluten-free products and raw materials needed for food preparation, with most participants having faced high barrier scores and found gluten-free products to be consistently expensive. This is a shared experience of celiac patients globally, and specifically Palestinians, given the difficult circumstances and economic constraints they face. Another factor that stood out is the boycotting of companies producing gluten-free products, which superimposed an additional layer of challenges on these patients, as all participants stated they could never find alternatives from non-boycotted manufacturers. However, one barrier that differed significantly between adherence groups is the stress and burden of meal preparation. Notably, higher adherence was significantly associated with not finding cooking to be a stressful task (*P* = 0.004). This aligned with broader research not specific to CD, highlighting a variety of positive outcomes related to home cooking, including healthier dietary patterns, better psychosocial outcomes, increased willingness to integrate complex dietary changes, and improved quality of life (QOL). Therefore, reframing gluten-free cooking from a burden into a manageable or even empowering routine appears to be a key psychological factor in promoting long-term dietary adherence and well-being^28^. As for social barriers, which revolved around the assessment of family/friends’ support, as well as navigating social situations, participants described social events such as weddings and family gatherings as high-risk scenarios for dietary relapses, and were associated with low adherence rates in these groups (*P* = 0.016). Direct interpersonal pressure may result in the intake of gluten-containing food upon forceful insistence of friends/family in such situations. In addition to that, the social norms to celebrate through food may directly contradict the private dietary discipline required for a gluten-free diet, challenging personal urge to adhere.

The results of our analysis were consistent with other previous research done within this area. However, what is novel in the current study is that when all of the factors, including CSI total score, GHQ total score, Total Awareness score, Attitude/Stance score, Access obstacles total, Social obstacles total, sex, age, monthly family income (≥ 3000 vs. < 3000), and smoking status were assessed simultaneously in the multivariable regression analysis, more positive attitudes toward the GFD remained the most consistent independent correlate of dietary adherence, whereas the association with male sex was less stable across sensitivity analyses. Across the sample, no significant associations were found between adherence and other sociodemographic factors, disease characteristics, awareness, and lifestyle.

Several limitations should be considered. First, dietary adherence was assessed using an investigator-developed questionnaire that was not formally validated against objective standards, raising the possibility of misclassification. Second, although internal consistency was assessed, test–retest reliability of the investigator-developed questionnaire domains was not evaluated because the questionnaire was administered only once. Third, the use of convenience sampling may have introduced selection bias and may limit the generalizability of the findings. Fourth, the cross-sectional design precludes causal inference and limits the interpretation of temporal relationships between adherence and associated factors. Fifth, because numerous bivariate comparisons were performed, the risk of type I error should be considered; therefore, bivariate findings not retained in adjusted or sensitivity analyses should be interpreted cautiously as exploratory. Finally, the relatively small sample size and the number of covariates included in the multivariable model may have increased the risk of model instability and overfitting, even though diagnostic analyses suggested acceptable calibration and discrimination.

## Conclusion

This study provides the first comprehensive analysis of gluten-free diet (GFD) adherence and its determinants among Palestinian adults with celiac disease. Adherence to a GFD among Palestinian adults with celiac disease remains suboptimal. While adherence is challenged by universal barriers such as the high cost and limited availability of gluten-free products, as well as stressful social situations, our multivariate analysis reveals that the core determinants in this population are psychosocial and perceptual rather than clinical or purely knowledge-based. Specifically, a more positive attitude toward the GFD emerged as the most consistent factor associated with higher adherence across adjusted and sensitivity analyses. The association with male sex was less stable and should be interpreted cautiously. Notably, factors like symptom burden, psychosocial distress, subjective knowledge, and barriers related to meal preparation and managing social situations, although initially significant in bivariate testing, did not retain an independent association with adherence after multivariable adjustment. Interventions must be reframed so that clinicians and educators strategically incorporate clear, evidence-based education about the serious long-term risks of non-adherence into routine celiac disease counseling, helping patients internalize the chronic severity of the condition, thereby fostering the positive attitude that our study has shown to be strongly linked with high adherence. In addition to that, addressing gender-specific burdens and structural economic barriers. Future research should employ longitudinal designs after successful counseling and objective adherence measures to clarify causality and explore the complex, bidirectional relationship between symptoms, motivation, and behavior.

## Supplementary Information

Below is the link to the electronic supplementary material.

Supplementary Material 1

## Data Availability

The de-identified participant-level dataset, the analysis code, and study materials are available from the corresponding author on reasonable request.
