# Supplementary material for: Factors associated with adherence to gluten-free diet among celiac patients in Palestine: a cross-sectional study
Source: Sci Rep. 2026 Apr 24;16:18929. doi: 10.1038/s41598-026-49948-4 (PMC13276410; doi:10.1038/s41598-026-49948-4)
Supplement: Supplementary file 1 — Supplementary Material 1 [file 41598_2026_49948_MOESM1_ESM.docx]

Data collection model

**Factors Associated with Adherence to Gluten-Free Diet Among Celiac Patients in Palestine: A cross-sectional Study**

(Saed. et al)

**Part One: Personal Information**

1. Gender : ☐ Male ☐ Female
2. Age: _______ years
3. Place of residence : ☐City ☐ Village ☐ Camp
4. marital status :

☐ Single​

☐ Married

☐ Other : ___________

1. Value of family income :

☐ Less than 1500

☐ From 1500 to 3000

☐ From 3000 to 5000

☐ More than 5000

1. Employment status :

☐ Worker / Employee

☐ Works part-time

☐ Retired

☐ Unemployed

1. Educational level :

☐ primary

☐ secondary

☐ University / Diploma

☐ Postgraduate Studies

**Part Two: Information related to disease diagnosis**

1. Age at diagnosis:
2. Number of years since diagnosis:
3. What prompted you to seek medical care that ultimately led to your diagnosis?
4. How was the diagnosis confirmed?
5. Laboratory tests
6. binoculars
7. Laboratory tests and endoscopies
8. Diagnosis by symptoms
9. Do you visit your doctor for regular follow-up appointmen. If yes, how many times a year?
   ☐ Yes  ☐ no
10. Do you visit a nutritionist for regular follow-up? If the answer is yes, how many times a year?
    ☐ Yes  ☐ no
11. Do you suffer from any other medical conditions? If the answer is yes, please state it.
12. Do you take any medications regularly? If the answer is yes, please state it.
13. Have you undergone any surgical procedures?? If the answer is yes, please state it.
14. Does anyone in your family have a diagnosis of celiac disease? If so, what is their relationship to you?

**Part Three: Information Related to the Disease**

1. Have you been bothered by pain or discomfort in the upper abdomen or the pit of the stomach during the past 4 weeks?
   1. Never
   2. rarely
   3. sometimes
   4. a lot
   5. always
2. Have you been bothered by nausea during the past 4 weeks?
   1. Never
   2. rarely
   3. sometimes
   4. a lot
   5. always
3. Have you been bothered by rumbling in your stomach during the past 4 weeks?
   1. Never
   2. rarely
   3. sometimes
   4. a lot
   5. always
4. Has your stomach felt bloated during the past 4 weeks?
   1. Never
   2. rarely
   3. sometimes
   4. a lot
   5. always
5. Have you been bothered by diarrhea during the past 4 weeks?
   1. Never
   2. rarely
   3. sometimes
   4. a lot
   5. always
6. When going on the toilet, have you had the sensation of not completely emptying your bowels during the past 4 weeks?
   1. Never
   2. rarely
   3. sometimes
   4. a lot
   5. always
7. Have you been bothered by hunger pains during the last 4 weeks?
   1. Never
   2. rarely
   3. sometimes
   4. a lot
   5. always
8. Have you been bothered by low energy levels during the past 4 weeks?
   1. Never
   2. rarely
   3. sometimes
   4. a lot
   5. always
9. Have you been bothered by headaches during the past 4 weeks?
   1. Never
   2. rarely
   3. sometimes
   4. a lot
   5. always
10. Have you had food cravings in the last 4 weeks?
    1. Never
    2. rarely
    3. sometimes
    4. a lot
    5. always
11. Have you had loss of appetite during the past 4 weeks?
    1. Never
    2. rarely
    3. sometimes
    4. a lot
    5. always
12. Related to Celiac Disease, how is your health?
    1. Excellent
    2. Good
    3. Medium
    4. bad
    5. Very bad
13. Overall, how is your health?
    1. Excellent
    2. Good
    3. Medium
    4. bad
    5. Very bad
14. How much physical pain have you had during the past 4 weeks?
    1. nothing
    2. a little
    3. middle
    4. a lot
    5. Too much
15. I am comfortable.
    1. I strongly agree
    2. I agree
    3. I neither agree nor disagree
    4. I oppose
    5. I strongly disagree
16. I am as healthy as anybody I know.
    1. I strongly agree
    2. I agree
    3. I neither agree nor disagree
    4. I oppose
    5. I strongly disagree
17. Do you have any other symptoms or concerns since being diagnosed with celiac disease that haven’t been addressed? If yes, please mention them.
    1. Yes
    2. no

**Part Four: Information Related to Compliance gluten-free diet**

**Section One: Self-Assessment of Knowledge**

1. Are you familiar with celiac disease (gluten intolerance): its symptoms, causes, and diagnosis?
   1. nothing
   2. Very few
   3. to some extent
   4. good
   5. Very large
2. How familiar are you with gluten-free products?
   1. nothing
   2. Very few
   3. to some extent
   4. good
   5. Very large
3. How well can you distinguish between gluten-free food and food containing gluten by reading the food label?
4. nothing
5. Very few
6. to some extent
7. good
8. Very large
9. How aware are you of the complications that may occur if you do not adhere to a gluten-free diet?
   1. nothing
   2. Very few
   3. to some extent
   4. good
   5. Very large

**Part Two: Awareness and Knowledge**

1. Which of the following statements about celiac disease is true? ) Table
   1. Celiac disease is an autoimmune disorder caused by consuming gluten . Yes/ No
   2. Its symptoms include diarrhea, bloating, and fatigue . Yes /No
   3. A gluten-free diet is the only effective treatment. Yes /No
   4. It can be treated by temporarily adhering to a gluten-free diet Yes/ No
   5. Occasionally consuming gluten does not harm celiac patients. Yes/No
   6. Celiac disease can be treated with medication. Yes No
   7. Celiac disease only affects the digestive system . Yes/No
   8. Failure to adhere to a gluten-free diet can lead to complications due to malabsorption, such as vitamin deficiencies, osteoporosis , and delayed growth in children . Yes No
   9. Failure to adhere to a gluten-free diet may cause serious long-term complications such as infertility and anemia. Yes/ No
2. Which of the following foods are naturally gluten-free? ( Choose all the correct options)
   1. rice
   2. barley
   3. potatoes
   4. wheat
   5. maize
3. Which of the following statements is true about gluten-free products? (Choose only one option )
   1. All gluten-free products are also milk-free .
   2. Gluten-free products can contain very small amounts of gluten that do not cause harm to most celiac patients .
   3. Gluten-free products cannot contain starches .
4. What are some common substitutes used in gluten-free products? ( Choose all correct options )
   1. almond flour
   2. rice flour
   3. Corn flour
   4. Oat flour (certified gluten-free )
   5. buckwheat flour
   6. Coconut flour
5. Which of the following statements indicates that the product is gluten-free? ( Option 1 )
   1. " Wheat-free "
   2. " Does not contain gluten ."
   3. Gluten -free and certified by [ official body ]
   4. " May contain traces of gluten ."
6. A gluten-free diet can be discontinued in a patient with celiac disease:
7. After the symptoms disappear
8. After improvements in laboratory tests and endoscopic examinations
9. After the symptoms disappeared and the laboratory tests improved Endoscopic examinations
10. It should not be stopped; it is for life.

**Section Three: The Stance**

1. Do you believe that adhering to a gluten-free diet is important for your health?
2. I strongly agree
3. I agree
4. neutral
5. I oppose
6. I strongly disagree
7. " The relief from symptoms motivates me to adhere to a gluten-free diet."
8. I strongly agree
9. I agree
10. neutral
11. I oppose
12. I strongly disagree
13. " Maintaining my long-term health encourages me to stick to a gluten-free diet ."
14. I strongly agree
15. I agree
16. neutral
17. I oppose
18. I strongly disagree
19. " I want to avoid the health complications resulting from consuming gluten, so I adhere to the diet ."
20. I strongly agree
21. I agree
22. neutral
23. I oppose
24. I strongly disagree
25. " Social motivation and family support help me stick to a gluten-free diet ."
26. I strongly agree
27. I agree
28. neutral
29. I oppose
30. I strongly disagree
31. A gluten-free diet actually helps in controlling symptoms.
32. I strongly agree
33. I agree
34. neutral
35. I oppose
36. I strongly disagree

**Section Four : Practices**

1. Do you read the ingredient labels on foods before buying or eating them?
2. never
3. sometimes
4. always
5. I check if the foods are labeled "gluten-free".
6. never
7. sometimes
8. always
9. How often do you think you are consuming gluten (intentionally or unintentionally) while following a gluten-free diet? This includes unintentional exposure to gluten exposure.
10. never
11. sometimes
12. always

1. I enjoy the taste of gluten-free foods .
2. never
3. sometimes
4. always
5. I eat my meals at home to reduce the risk of consuming gluten.
6. never
7. sometimes
8. always
9. Be sure to use special cooking utensils and cookware to avoid food contamination with gluten.
10. never
11. sometimes
12. always
13. I avoid eating any food if I am not sure it is gluten-free :

a. Never

b. Sometimes

c. Always

**Part Five: Information Related to Obstacles Adhering to a gluten-free diet**

**First: Access and Availability**

1. I find it difficult to find gluten-free products on the market.
2. never
3. sometimes
4. always
5. "I have difficulty finding a variety of gluten-free food options."
6. never
7. sometimes
8. always
9. Do you find gluten-free products expensive?
   1. never
   2. sometimes
   3. always
10. Do you consider cooking gluten-free foods to be stressful or a burden?
    1. never
    2. sometimes
    3. always
11. I find it difficult to prepare gluten-free meals at home due to the difficulty in obtaining raw materials.
12. never
13. sometimes
14. always
15. I find it difficult to prepare gluten-free meals at home due to the difficulty of handling raw materials:
16. n
17. sometimes
18. always
19. I faced difficulty in preparing gluten-free meals at home because the raw materials were expensive:
20. never
21. sometimes
22. always
23. Do you receive sufficient support from restaurant staff to provide gluten-free meals?
24. never
25. sometimes
26. always
27. " I find it difficult to find gluten-free products on the market that were produced by companies that are not boycotted ."
28. never
29. sometimes
30. always

**Second: Social obstacles**

1. Are your friends or family members aware of celiac disease ?
2. never
3. sometimes
4. always
5. Do your friends or family encourage you to adhere to a gluten-free diet?
   1. never
   2. sometimes
   3. always
6. Do you consume gluten because of social pressure around you?
7. never
8. sometimes
9. always
10. Can you resist the temptation to eat gluten-containing foods at family gatherings or weddings?
11. never
12. sometimes
13. always
14. I feel socially isolated because of my special diet.
15. never
16. sometimes
17. always
18. I feel embarrassed telling others about my health condition.
19. never
20. sometimes
21. always
22. Do you smoke?

If you are a current smoker or were a former smoker, how many years have you been smoking?

**Part Six: Lifestyle Information**

1. How many hours do you sleep per night (on average)?
   _______ hours
2. Do you suffer from any sleep-related problems?
    ☐ Yes  ☐ no
    If the answer is yes, please explain : _____________________________
3. Choose from the following what applies to your daily physical activity :
4. Walking regularly inside the house
5. Walking regularly outside the house
6. Regular exercise at home
7. Regular exercise at gyms
8. I do not engage in any physical activity

**Part Seven: Information Related to Mental Health**

1. Have you recently been able to concentrate on whatever you’re doing?
   a. Better than usual
   b. Same as usual
   c. Less than usual
   d. Much less than usual
2. Have you recently lost much sleep over worry?
   a. Not at all
   b. Not more than usual
   c. More than usual
   d. Much more than usual
3. **Have you recently felt that you are playing a useful part in things?**
   a. More than usual
   b. Same as usual
   c. Less than usual
   d. Much less than usual
4. Have you recently felt capable of making decisions about things?
   a. More than usual
   b. Same as usual
   c. Less than usual
   d. Much less than usual
5. Have you recently felt constantly under strain?
   a. Not at all
   b. Not more than usual
   c. More than usual
   d. Much more than usual
6. Have you recently felt you couldn’t overcome your difficulties?
   a. Not at all
   b. Not much more than usual
   c. More than usual
   d. Much more than usual
7. Have you recently been able to enjoy your normal day-to-day activities?
   a. More than usual
   b. Same as usual
   c. Less than usual
   d. Much less than usual
8. Have you recently been able to face up to your problems?
   a. More than usual
   b. Same as usual
   c. Less than usual
   d. Much less than usual
9. Have you recently felt unhappy and depressed?
   a. Not at all
   b. Not more than usual
   c. More than usual
   d. Much more than usual
10. **Have you lost confidence in yourself?**
    a. Absolutely
    b. Not much more than usual
    c. More than usual
    d. Much more than usual
11. Have you recently felt that you are worthless?
    a. Not at all
    b. Not much more than usual
    c. More than usual
    d. Much more than usual
12. Did you feel somewhat happy?
    a. More than usual
    b. About the same as usual
    c. Less than usual
    d. Much less than usual
